# Supplementary material for: The interactome of intact mitochondria by cross-linking mass spectrometry provides evidence for coexisting respiratory supercomplexes
Source: Mol Cell Proteomics. 2017 Dec 8;17(2):216–32. doi: 10.1074/mcp.RA117.000470 (PMC5795388; doi:10.1074/mcp.RA117.000470)
Supplement: Supplemental Data [file supp_RA117.000470_133922_0_supp_23978_fzffwf.zip › spectra_annotation/mito_DR_spectra_annotation/78-1-1-1-17-1.pdf]

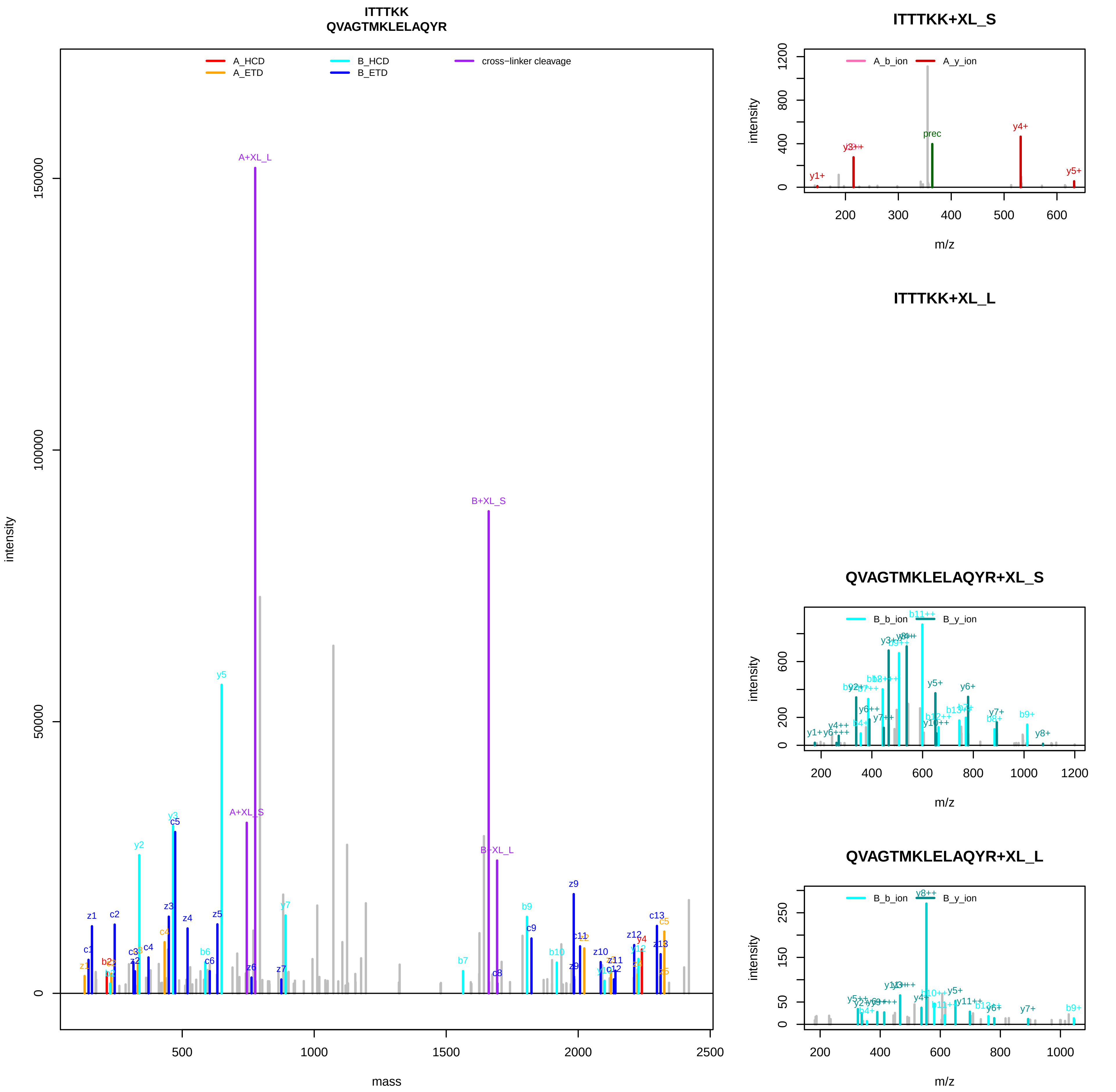
ITTTKK
QVAGTMKLELAQYR
A_HCD
A_ETD
B_HCD
B_ETD
cross−linker cleavage
A+XL_L
B+XL_S
A+XL_S
B+XL_L
y5
y3
c5
y2
z1
c1
c2
b2
c3
c4
z2
z3
z4
c4
b6
c6
z5
z6
z7
y7
b7
c8
b9
c9
b10
z9
c11
z10
c12
z11
z12
y4
c13
z13
c5
intensity
mass
0
50000
100000
150000
500
1000
1500
2000
2500
ITTTKK+XL_S
A_b_ion
A_y_ion
y1+
y3++
prec
y4+
y5+
intensity
m/z
0
400
800
1200
200
300
400
500
600
ITTTKK+XL_L
QVAGTMKLELAQYR+XL_S
B_b_ion
B_y_ion
b11++
y5+
y6+
y7+
b9+
y8+
y1+
y4++
y6+++
y6++
y7++
y10++
b8+
intensity
m/z
0
200
600
200
400
600
800
1000
1200
QVAGTMKLELAQYR+XL_L
B_b_ion
B_y_ion
y8++
y5+
y11++
y6+
y7+
b9+
y4+
intensity
m/z
0
50
150
250
200
400
600
800
1000
